# Impact of positive end-expiratory pressure on renal resistive index in mechanical ventilated patients

**DOI:** 10.1007/s10877-024-01172-z

**Published:** 2024-05-21

**Authors:** Alberto Fogagnolo, Salvatore Grasso, Elena Morelli, Francesco Murgolo, Rosa Di Mussi, Luigi Vetrugno, Riccardo La Rosa, Carlo Alberto Volta, Savino Spadaro

**Affiliations:** 1 Department of Translational medicine, Azienda Ospedaliera-Universitaria Sant’ Anna, University of Ferrara, 8, Aldo Moro 44121, Ferrara, Italy; 2 Dipartimento dell’Emergenza e Trapianti d’Organo (DETO), Sezione di Anestesiologia e Rianimazione, Università degli Studi di Bari “Aldo Moro”, Bari, Italy; 3 Intensive Care Unit, Department of Surgery, Dentistry, Maternity and Infant, University and Hospital Trust of Verona, Verona, Italy; 4 Department of Medical, Oral and Biotechnological Sciences, University of Chieti-Pescara, Chieti, Italy

**Keywords:** Positive end-expiratory pressure, Renal hemodynamics, Acute kidney injury, Doppler ultrasound, Critical care

## Abstract

Purpose: Growing evidence shows the complex interaction between lung and kidney in critically ill patients. The renal resistive index (RRI) is a bedside measurement of the resistance of the renal blood flow and it is correlated with kidney injury. The positive end-expiratory pressure (PEEP) level could affect the resistance of renal blood flow, so we assumed that RRI could help to monitoring the changes in renal hemodynamics at different PEEP levels. Our hypothesis was that the RRI at ICU admission could predict the risk of acute kidney injury in mechanical ventilated critically ill patients. Methods: We performed a prospective study including 92 patients requiring mechanical ventilation for ≥ 48 h. A RRI ≥ 0.70, was deemed as pathological. RRI was measured within 24 h from ICU admission while applying 5,10 and 15 cmH_2_O of PEEP in random order (PEEP trial). Results: Overall, RRI increased from 0.62 ± 0.09 at PEEP 5 to 0.66 ± 0.09 at PEEP 15 (*p* < 0.001). The mean RRI value during the PEEP trial was able to predict the occurrence of AKI with AUROC = 0.834 [95%CI 0.742–0.927]. Patients exhibiting a RRI ≥ 0.70 were 17/92(18%) at PEEP 5, 28/92(30%) at PEEP 10, 38/92(41%) at PEEP 15, respectively. Thirty-eight patients (41%) exhibited RRI ≥ 0.70 at least once during the PEEP trial. In these patients, AKI occurred in 55% of the cases, versus 13% remaining patients, *p* < 0.001. Conclusions: RRI seems able to predict the risk of AKI in mechanical ventilated patients; further, RRI values are influenced by the PEEP level applied.

**Trial registration**: Clinical gov NCT03969914 Registered 31 May 2019.

**Supplementary Information:**

The online version contains supplementary material available at 10.1007/s10877-024-01172-z.

## Introduction

Lung and kidney injuries are common in critically ill patients and frequently they are concomitant. The crosstalk between kidney and lung has not yet been fully elucidated in mechanically ventilated patients [1]. Indeed, patients admitted to intensive care unit (ICU) for acute respiratory failure are at an increased risk of acute kidney injury (AKI). The risk of AKI is increased threefold in patients needing mechanical ventilation [2, 3], thus coining the definition of “ventilator-induced kidney injury” [4]. 

Regarding hemodynamics, mechanical ventilation impairs kidney function mainly by decreasing cardiac output and increasing intrathoracic pressure [5–7]. The latter results in systemic venous congestion that may increase renal interstitial pressure, leading to decreased glomerular filtration rate (GFR). Both these mechanisms are magnified by the effects of positive end-expiratory pressure (PEEP), commonly applied in critically ill patients to improve oxygenation, reduce atelectasis [8, 9], decrease driving pressure [10] and minimize tidal reopening-collapse of unstable lung regions (atelectrauma) [11].

The doppler-based renal resistive index (RRI) has been proposed as a rapid, repeatable and noninvasive tool that allows the evaluation of renal hemodynamics through the analysis of flow velocities through the renal arterioles obtained by pulsed Doppler ultrasonography and able to identify the risk of AKI in postoperative and critically ill patients [12–16]. Several studies suggested that RRI could be useful to predict the renal outcome in critically ill patients with shock [17, 18] and COVID-19 [19, 20]. However, it was not confirmed in a recent multicentre study in mechanically ventilated patients [21]. Nowadays, the RRI is not only considered as a measure of renal vascular resistance, but rather reflects the hemodynamic both of the renal microcirculation and systemic circulation.

Renal resistive index has a relationship with the renal vascular resistances but may also be influenced by confounding factors, such as renal interstitial pressure, vascular compliance and intra-abdominal pressure, which may overall mitigate its ability in predicting the risk of AKI [15, 16]. 

RRI higher than 0.70 may represent a reasonable cut-off value and most commonly used to identify to predict a worsen outcome in critically ill patients [12, 13, 19, 22]. Of note, pathological RRI values (i.e., ≥ 0.70) are associated with arterial vasoconstriction, renal congestion, increase in interstitial pressure and glomerular capillary rarefaction (i.e. decreased vascular density caused by vasoconstriction-related ischemia) [23, 24]. 

In the clinical practice in ICU, mechanically ventilated patients require respiratory optimization by changes in PEEP but often the choice is measured as only in terms of improvement in oxygenation and respiratory compliance, disregarding possible deleterious effects on the kidney. The impact of PEEP on renal hemodynamics has seldom been assessed [25] and little is known on how to monitor this issue at bedside.

We hypothesized that the RRI could predict the development of AKI in mechanically ventilated critically ill patients with acute respiratory failure. In addition, we assumed that the RRI changes during a PEEP trial could help to identify patients at higher risk of PEEP-induced changes in renal hemodynamics.

Thus, we investigated the ability of the RRI obtained to predict the development of AKI in the 5 days following the ICU admission. Furthermore, we described the changes in RRI values measured at three different PEEP levels (5, 10 and 15 cmH_2_O).

## Methods

### Patients

This prospective study was conducted in the department of Intensive Care at the University Hospital of Ferrara, (Italy) from June 2019 to June 2021. The study was approved by local ethic committees (Approval number n° EM612-2019-276. Date of approval 21th May 2019. Board name: Comitato Etico di Area Vasta Emilia Centro della Regione Emilia-Romagna). Procedures were followed in accordance with the ethical standards of the institutional responsible committee on human experimentation and with the Helsinki Declaration of 1975. We included in the study, consecutive patients admitted to ICU expected to be mechanically ventilated for at least 48 h. Exclusion criteria were: age < 18 years old, patients with diagnosis of acute kidney injury of any stage before ICU admission, patients with history of previous renal replacement therapy, inadequate ultrasound view of the kidneys, arrhythmias (i.e. atrial fibrillation), and pregnancy. Enrollment of patients was stopped from January 2020 to May 2020 when our ICU was fully dedicated to the treatment of SARS-CoV-2 patients. Written informed consent was obtained from all patients able to give or next of kin.

### Data collection

Demographical data, including age, sex and body mass index were recorded at admission together with Simplified Acute Physiology Score (SAPS) II, Sequential Organ Dysfunction Score Assessment (SOFA), reasons for ICU admission and comorbidities. Acute respiratory failure was determined when ventilatory support with intubation and/or positive airway pressure was needed to avoid hypoxemia and/or hypercapnia. Chronic kidney disease (CKD) was defined according to Kidney Disease Improving Global Outcomes (KDIGO) guidelines [26]. Patients with CKD were included in the study if their serum creatinine on admission does not meet the criteria of AKI when compared to baseline. Daily serum creatinine and urinary output were collected from start date of inclusion for a total of seven days. Blood gas analysis (BGA) were obtained with a GEM Premier™ 4000 (Werfen, Le Pré-Saint-Gervais, France).

### Study protocol

On the first day of mechanical ventilation, patients were included in the study and underwent three RRI measurements during a PEEP trial (i.e. 5,10 and 15 cmH_2_O, applied in random order). RRI was measured according to previous studies. [17, 27–28] In the events of hemodynamic instability (defined as a 20% fall of mean blood pressure compared to baseline and/or occurrence of heart rate < 40 or > 140 bpm), the PEEP trial was stopped and the patient was considered as lost to follow-up. During the PEEP trial, patients were deeply sedated and ventilated with volume-controlled ventilation, with a tidal volume (Vt) of 6–7 ml/kg of predicted body weight. After the trial, the clinical PEEP level was resumed. Clinical PEEP was chosen by the threating physician; as a standard protocol in our department, PEEP level is usually set in order to minimize the driving pressure of respiratory system (ΔP) with a target of ΔP below 14 cmH_2_O. In case of ΔP persistently equal or above 14 cmH_2_O after PEEP titration, a further reduction of TV is adopted. Each level of PEEP was maintained for 15 min in order to allow the effects of PEEP to reach an equilibrium [29, 30]. At each PEEP level, blood gas analysis, mean arterial pressure and respiratory mechanics measurements, need for vasopressor drugs and fluid responsiveness were collected.

The end-inspiratory plateau pressure was obtained with an end-inspiratory occlusion of 3–5 s, while the total PEEP (PEEP_tot_) was obtained with an end-expiratory occlusion of 3–5 s. Driving Pressure (ΔP) was calculated as plateau pressure – PEEP_tot_; static respiratory system compliance was calculated as Vt/(end-inspiratory plateau pressure - PEEP_tot_).

Fluid responsiveness was defined as a 5% increase in the value of end-tidal carbon dioxide (ETCO_2_) during a passive leg raising (PLR) test [31, 32] or as a pulse pressure variation > 13% (when performing PLR was contraindicated).

Occurrence of AKI was defined according to KDIGO guidelines (i.e. an increase in serum creatinine of 0.3 mg/dl within 48 h or an increase in serum creatinine to 1.5 times the baseline value present within the previous 7 days, or urine volume < 0.5 ml/kg/h for 6 h) [33]. All patients received the same strategies to prevent the occurrence of acute kidney injury, including avoiding hypo/hypervolemia, avoiding nephrotoxic drugs when possible, glycemic control according to guidelines, infection source control and targeting hemodynamic stability.

In a subgroup of patients, cardiac output (CO) was calculated through trans-thoracic echography as the product of the heart rate, left ventricular outflow tract (LVOT), velocity time integral (VTI), and the area of the outflow tract as follows:

CO= (heart rate) (LVOT VTI) (π) (outflow tract diameter/2)^2^ [26]. 

### RRI measurement

RRI was measured in semi-recumbent position through a 5 MHz pulsed-wave Doppler probe (Edge II, SonoSite, Inc., USA) in the right kidney, except in cases of unsatisfactory image quality on the right side [17, 27–28] and was performed on site by the two investigators (AF and EM), both blind with regards to the level of PEEP applied during the measurement. The clinician in charge, not involved in the assessment, modify randomly the PEEP level in according to the protocol. After visualizing the kidney with a posterolateral approach, an interlobar or arcuate artery was selected for pulse wave Doppler measurements. At least three recordings were obtained from the selected arteries, and the mean value was used for the analysis. RRI was calculated as: (peak systolic velocity-end diastolic velocity)/peak systolic velocity). Peak systolic velocity and end diastolic velocity assessment was performed using the same waveform.

Values of RRI < 0.7 were considered as normal [18, 34–36]. Each level of PEEP was maintained for 15 min in order to allow the effects of PEEP to reach an equilibrium [29, 30]. At each PEEP level, blood gas analysis, arterial mean pressure and respiratory mechanics measurements, need for vasopressor drugs and fluid responsiveness were collected.

### Outcomes and subgroup analysis

The main outcome was to describe the ability of RRI to predict the risk of AKI in mechanically ventilated patients at ICU admission. Furthermore, we explored the impact of a PEEP trial on RRI and its ability to predict the occurrence of AKI. The secondary outcome was to describe changes in RRI at each PEEP step. We elucidated whether patients presenting a high renal resistive index (≥ 0.70) at least once during the PEEP trial could have an increased risk of AKI and verified if patients ventilated with a clinical PEEP higher than the PEEP level associated with RRI > 0.70 during the PEEP trial experienced an increased risk of AKI.

Subgroup analyses were also performed to investigate the relationship between hypovolemia and PEEP-induced changes in renal hemodynamics, and to ascertain if PEEP-induced changes in RRI were correlated to fluid responsiveness. In the sub-group of patients in which CO was determined, we investigated the relationship between PEEP-induced changes in CO and RRI. Finally, we investigated the relationship between RRI and PEEP-induced variation in lung mechanics.

### Statistical analysis

All the analysis reported were pre-planned unless specifically reported. Normal distribution of data was tested by the Shapiro–Wilk Normality Test. Data were reported as mean ± standard deviation or median [interquartile range] when appropriate. Pearson chi-square (*x*²) test was used for categorical data. Unpaired Student’s t-tests or Mann–Whitney U-tests were used for data with normal or non-normal distribution, respectively. PEEP-induced variations in RRI were analyzed with repeated measure ANOVA. Area under the curve (AUC) with 95% confidence of interval was used to analyze the ability to predict the occurrence of AKI. Specificity, sensibility, positive predictive value and negative predictive value were also calculated. Patients were analyzed in two groups, depending on the occurrence or not of AKI during the ICU stay. Multivariable logistic regression models were performed to investigate the AKI predictors; we included as covariate in the model all the variables with *p* ≤ 0.10 at univariate analysis. Multicollinearity was measured by variance inflation factors (VIF) and tolerance was set at VIF < 5. Intraclass correlation (ICC) analysis was used to assess the agreement in RRI measurements between the two investigators.

P-value ≤ 0.05 was considered statistically significant. Statistical analyses were performed using SPSS Statistics for Windows, Version 20.0 (IBM, Armonk, NY, USA).

### Sample size

Given a predicted AUROC of 0.720 [37] and an anticipated 19% occurrence of AKI [38], we planned to analyze 89 patients to achieve a power of 80% and alfa error of 5%. Considering a 15% loss to follow-up, we finally enrolled 105 patients.

## Results

### Population

During the study period, 105 patients were enrolled. Of those, 13 did not complete the study (10 due to unsatisfactory ultrasound imaging, 3 due to hemodynamic instability during the PEEP trial), leaving 92 patients for the final analysis. The flowchart of the study is shown in Supplement. Demographic and clinical characteristics of patients at ICU admission are resumed in Table 1. There were no significant differences in terms of the use of Angiotensin-converting enzyme and Angiotensin receptor blockers between the two groups of patients.

**Table 1:** Clinical and demographical characteristics of patients. Data are reported as mean±SD or median [IQR] as appropriate. BMI= Body mass index; COPD= Chronic obstructive pulmonary disease; ACE= Angiotensin-converting enzyme; ARB= Angiotensin receptor blockers; PEEP= Positive end-expiratory pressure; ICU= Intensive care unit; CRRT= Continuous renal replacement therapy

Variables at admission	All patients (*n*=92)	Occurrence of AKI (*n*=28)	Patients without AKI (*n*=64)	*P* value
Age, years	72 ± 12	76 ± 8	67 ± 12	0.001
SAPS II score	38±12	40±10	36 ± 11	0.1
SOFA score at admission	5 [3 – 7]	6 [3–8]	5 [3–7]	0.81
BMI, kg/m^2^	29 ± 7	29 ± 6	29 ± 8	0.86
Diabetes, n (%)	18 (19)	8 (29)	10 (16)	0.164
Hypertension, n (%)	57 (62)	19 (68)	38 (59)	0.491
History of cardiac disease, n (%)	42 (46)	13 (46)	29 (45)	0.999
COPD, n (%)	20 (22)	8 (29)	12 (19)	0.41
Chronic kidney disease, n (%)	21 (23)	10 (36)	11 (17)	0.063
ACE inhibitors or ARB prior to admission, n (%)	42 (46)	14 (50)	28 (44)	0.579
*Reason for ICU*				
Sepsis/Septic shock, n (%)	36 (39)	12 (43)	24 (37)	0.801
Respiratory failure n (%)	35 (38)	11 (39)	24 (37)	0.871
- *ARDS*	28 (30)	8 (28)	20 (31)	
Cardiogenic shock, n (%)	9 (10)	3 (11)	6 (9)	0.842
Hypovolemic shock, n (%)	10 (11)	2 (7)	8 (12)	0.694
Trauma, n (%)	2 (2)	/	2 (3)	0.865
*Laboratory data at ICU admission*				
Hemoglobin, g/dL	11.0 ± 2.4	11.4 ± 1.9	11.4 ± 1.9	0.945
Baseline creatine, mg/dL	1.14 [0.82 – 1.96]	1.47 [0.95 – 3.13]	0.99 [0.79 – 1.61]	0.002
*Clinical data at ICU admission*				
Need for vasopressor, n (%)	36	11	25	0.999
*Norepinephrine, mcg/Kg/min*	*0.289 ± 0.2*	*0.32 ± 0.2*	*0.26 ± 0.2*	*0.51*
Mean arterial pressure, mmHg	79 ± 12	76 ± 12	80 ± 12	0.184
Heart rate, bpm	81 ± 21	82 ± 18	81 ± 20	0.948
PEEP, cmH_2_O	8 [6 – 10]	8 [6 – 10]	8 [6 – 12]	0.574
Fluid responsiveness, n (%)	38 (41)	12 (43)	26 (41)	0.51
*Outcomes*				
72-h Fluid balance, ml	670 [320-1230]	1480 [600–2600]	625 [225-880]	<0.001
ICU mortality, n (%)	22 (24)	10 (36)	12 (19)	0.111
Need for CRRT	4 (4)	4 (16)	/	0.011

During the first 5 days of ICU stay, AKI occurred in 30% (28/92) of the patients. Among patients with AKI, Stage 1 AKI was detected in 21/28 (72%) patients, whereas 2/28 (7%) had stage 2 AKI and 6/28 stage 3 AKI (21%). Four of them needed renal replacement therapy (RRT) during their ICU stay (5%). Daily changes in creatinine values in AKI and not-AKI patients are summarized in Supplemental Fig. 1. At the first day of ICU stay, age and baseline creatinine were higher in AKI patients (Table 1).

**Fig. 1:**
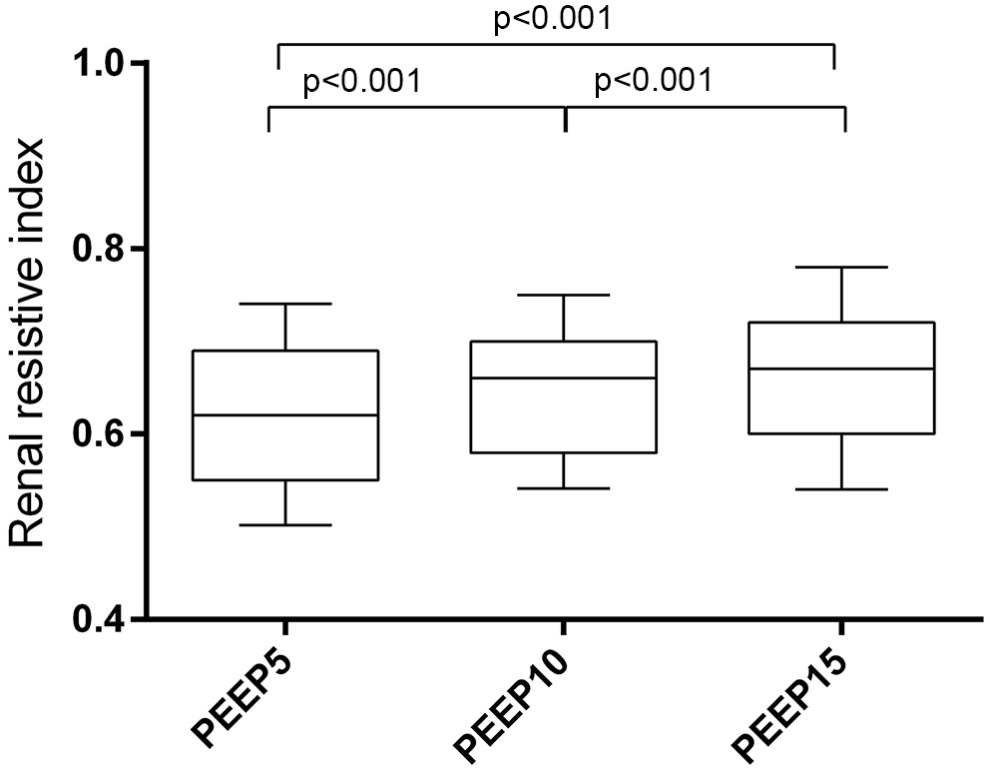
Renal resistive index values at different levels of PEEP

The RRI increased significantly (Table 2) from 0.62 ± 0.09 at PEEP 5 to 0.66 ± 0.09 at PEEP 15 (*p* < 0.001). (Fig. 1) Patients exhibiting a pathological RRI (defined as RRI ≥ 0.70) were 17/92 (18%) at PEEP 5, 28/92 (30%) at PEEP 10, 38/92 (41%) at PEEP 15, respectively. Intraclass correlation showed a very good agreement among the two appraisers with an ICC value of 0.991 [0.986–0.994]. Thirty-eight patients (41%) exhibited RRI ≥ 0.70 at least once during the PEEP trial. In these patients AKI occurred in 55% of the cases, whereas in the remaining patients the incidence of AKI was 13%, *p* < 0.001.

**Table 2:** Study variables at each PEEP level in AKI and not-AKI patients

Variables	PEEP 5 cmH_2_O	PEEP 10 cmH_2_O	PEEP 15 cmH_2_O	*p* value for inter-group trend	*p* value for group trend comparison
*Renal resistive index*					
All patients	0.62 ± 0.09	0.65 ± 0.08	0.66 ± 0.09	<0.001	
AKI	0.69 ± 0.08	0.73 ± 0.07	0.74 ± 0.07	<0.001	0.53
Not AKI	0.59 ± 0.07	0.62 ± 0.07	0.63 ± 0.07	<0.001	
*Mean arterial pressure*					
All patients	80 [68 – 87	78 [68 – 85]	77 [68 – 85]	0.017	
AKI	77 [68 – 83]	77 [63 – 85]	76 [62 – 81]	0.73	0.79
Not AKI	80 [70 – 89]	78 [70 – 89]	77 [69 – 87]	0.01	
*PaO*_*2*_ */F*_*I*_ *O*_*2*_					
All patients	229 [162 – 321]	230 [177 – 303]	241 [182 – 296]	0.17	
AKI	250 [162 – 361]	238 [181 – 320]	263 [241 – 286]	0.09	0.93
Not AKI	225 [158 – 321]	228 [176 – 298]	229 [170 – 310]	0.67	
*PaCO2*					
All patients	43 ± 10	43 ± 10	44 ± 11	0.51	
AKI	43 ± 8	42 ± 6	45 ± 9	0.48	0.18
Not AKI	43 ± 10	43 ± 10	44 ± 11	0.49	
*Lactate, mmol/L*					
All patients	1.5 [1.0 – 2.5]	1.6 [1.1 – 2.6]	1.4 [1.1 – 2.0]	0.63	
AKI	1.8 [1.2 – 4.6]	1.8 [1.2 – 4.1]	1.4 [1.2 – 2.8]	0.86	0.69
Not AKI	1.5 [1 – 2.4]	1.5 [1 – 2.3]	1.4 [1.1 – 2.0]	0.72	
*Plateau pressure*					
All patients	14 ± 4	19 ± 4	24 ± 3	<0.001	
AKI	14 ± 5	19 ± 5	24 ± 4	<0.001	0.91
Not AKI	14 ± 3	19 ± 3	24 ± 3	<0.001	
*Driving pressure*					
All patients	10 ± 3	9 ± 2	9 ± 2	0.049	
AKI	11 ± 4	10 ± 3	10 ± 4	0.85	0.72
Not AKI	10 ± 3	9 ± 2	9 ± 3	0.47	
*Respiratory rate*					
All patients	15 [14 – 18]	15 [14 – 18]	15 [14 – 20]	0.77	
AKI	15 [14 - 17]	15 [14 – 17]	15 [12 – 17]	0.79	0.66
Not AKI	15 [13 – 18]	15 [13 – 20]	15 [14 – 20]	0.95	

**Fig. 2:**
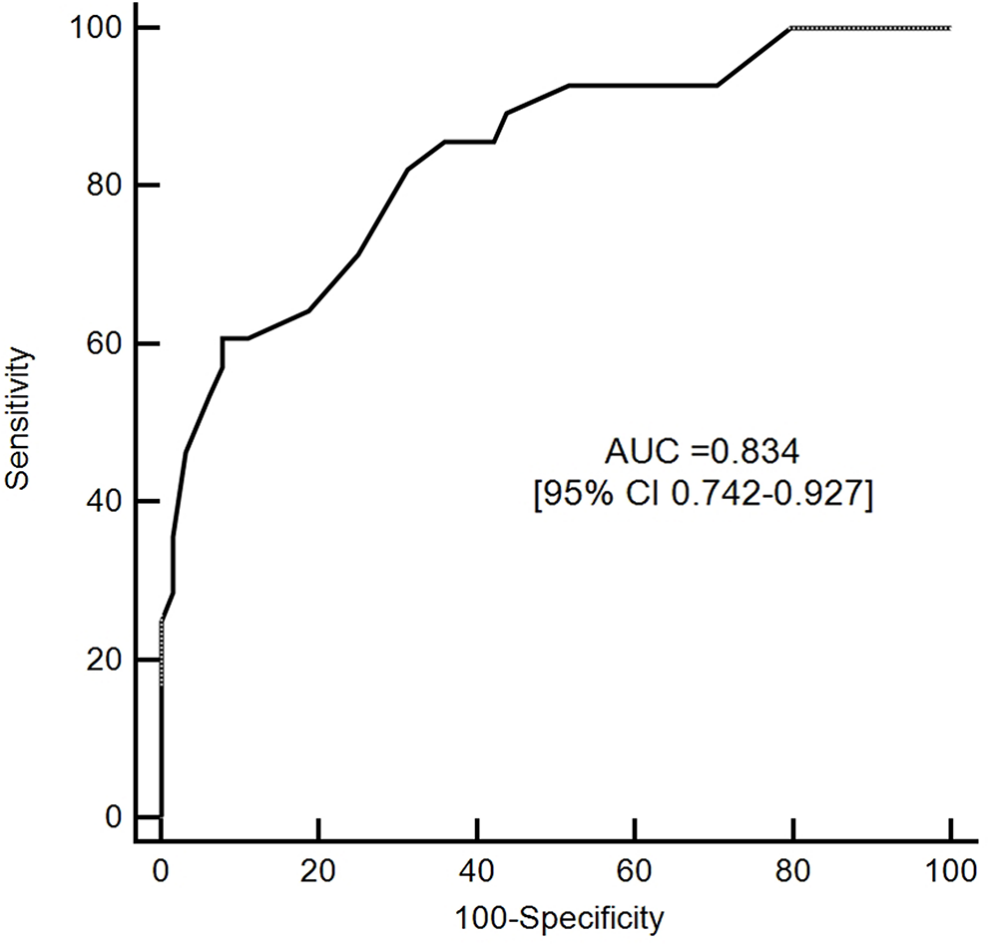
ROC curve analysis of the ability of the mean value of RRI obtained during the PEEP trial to predict AKI

The mean RRI value during the PEEP trial was able to predict the occurrence of AKI with AUROC = 0.834 [95% CI 0.742–0.927]. Specificity and sensibility were 92.2 and 60.7, respectively, with a positive predictive value of 77% and a negative predictive value of 85%. (Fig. 2) The positive Likehood ratio for RRI ≥ 0.70 was 7.7 [95% CI 3.2–19.0]. The predictive value of the RRI measured at each level of PEEP is shown in Supplement and in Supplemental Fig. 2.

Furthermore, we performed a post-hoc analysis in 44 patients in which RRI was measured at clinical PEEP level; the AUROC of RRI measured at clinical PEEP for detecting AKI was 0.830 [95% CI 0.68–0.98].

**Fig. 3:**
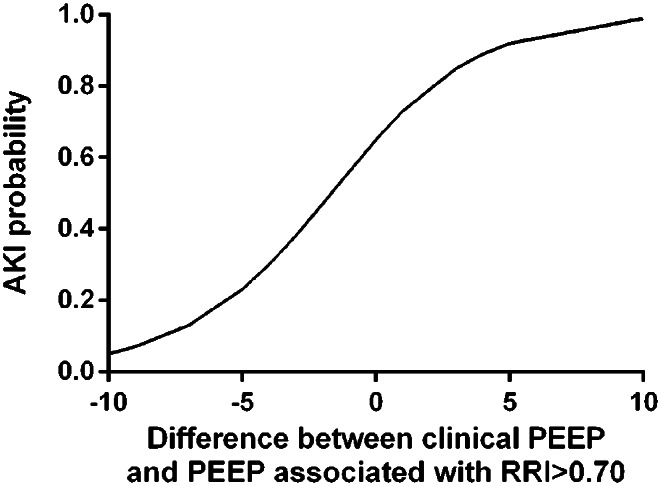
Relationship between the clinical PEEP and the probability of AKI. The *x* axis describes the difference between the clinical PEEP and the PEEP associated with RRI>0.70 during the PEEP trial. For example, if a clinical PEEP of 12 cmH2O was chosen and a pathological RRI was detected at PEEP 10, a value of “2” was indicated in the *x* axis

The clinical PEEP level was not different between patients who developed AKI and patients who did not (8 [6–10] cmH_2_O vs. 8 [6–12] cmH_2_O, *p* = 0.67) (Table 1). Of note, patients ventilated with a clinical PEEP level higher or equal than the one associated with a pathological RRI during the PEEP trial, had increased risk of AKI. (Fig. 3, Supplemental Fig. 3). A clinical PEEP higher than the PEEP associated with RRI > 0.70 was independently associated with subsequent development of AKI (Supplemental Table 1).

When analyzing the respiratory mechanics at each PEEP levels, ∆P decreased from PEEP 5 to PEEP 10 (*p* = 0.03), a further decrease between PEEP 10 and PEEP 15 was not identified. (Table 2). The ∆P and the plateau pressure trends did not differ between patients with or without RRI ≥ 0.70 at each PEEP level.

### Predictors of RRI

With reference to hemodynamic parameters, we found a weak significant correlation only between diastolic pressure and RRI at each PEEP value (*r*=-0.20 at PEEP 5, *r*=-0.21 at PEEP 10, *r*=-0.25 at PEEP 15). In the cohort of patients where CO was assessed during the PEEP trial, CO did not differ between patients presenting a RRI ≥ 0.70 compared to patients with normal RRI values throughout the PEEP trial. (Table S2)

## Discussion

The main findings of our study are that the RRI value can predict the occurrence of AKI and that the PEEP levels can affect renal hemodynamics (as assessed by the RRI itself) with a non-linear effect considerably different between patients. Further, patients presenting at least once a RRI higher than normal (i.e. ≥ 0.70) during a PEEP trial performed at ICU admission developed significantly more AKI in the following five days. Interestingly, patients ventilated with a clinical PEEP level higher or equal than the one associated with a pathological RRI, had increased risk of AKI.

The occurrence of AKI in mechanically ventilated patients is associated with worse outcome and patients with combined AKI and respiratory failure have a mortality rate as high as 50%, even in case of mild AKI [39]. Overall, our data suggest that some patients may be predisposed to PEEP-induced impairment of renal perfusion and that a PEEP trial could identify them at ICU admission. The relationship between PEEP and AKI is still debated. Whereas a meta-analysis did not show any relationship between PEEP and AKI [2], recent studies found a 5-fold increase in the risk of AKI in patients ventilated with PEEP above 14 cm H_2_O [40] and showed that PEEP is the only ventilator parameter independently correlated with AKI [41]. Several physiological mechanisms may explain the effects of PEEP on kidney function. *First*, PEEP increases central venous pressure (CVP) [42] by increasing the intrathoracic pressure as shown by Shojaee and coworkers, that found a linear correlation between PEEP and central venous pressure (CVP) [43]. Since venous vascular resistances are negligible, CVP is easily transmitted backward to the renal veins increasing the renal interstitial hydrostatic pressure [44] and the backward resistance to glomerular outflow [42]. It reasonable to speculate that such hemodynamic effect may increase the RRI [27]. A recent meta-analysis confirmed the correlation between higher CVP and the risk of AKI [44]. However, a significant number of patients does not experience AKI even at higher PEEP-induced CVP levels [45]. Another putative mechanism could be a PEEP-induced decrease in cardiac output. However, we measured CO in a sub-group of 28 patients during the PEEP trial and we were not able to find any correlation between CO and RRI. These data seem to confirm a previous study by Oliveira et al. showing no relationship between CO and RRI [28]. Indeed, according to classical physiological studies, as CO decreases more blood is directed to the kidneys. Thus, at least in the early phases, renal hemodynamics is relatively preserved or only slightly impaired [46] and this adaptive effect could have masked the effect of our PEEP trial on renal perfusion and hence on the RRI.

Our data suggest that evaluating the response of RRI to a PEEP trial could be a promising diagnostic tool to predict the risk of AKI in mechanically ventilated patients, in particular when we try to combine lung protection and kidney function. We found that among the 38 patients who showed at least once an RRI ≥ 0,70 during the PEEP trial, those ventilated with a clinical PEEP equal or higher than the PEEP level associated with an RRI ≥ 0.70 at the PEEP trial (21/38), developed AKI in 90% of the cases (19/21) whereas those ventilated with a clinical PEEP lower than the PEEP level associated with an RRI ≥ 0.70 at the PEEP trial (17/38) developed AKI in 12% of the cases (2/17) (*p* < 0.001). Supplemental Fig. 2 shows the occurrence of AKI as a function of the difference between the clinical PEEP and the lowest PEEP level associated with an RRI ≥ 0.70 at the PEEP trial. The difference between the clinical PEEP and the lowest PEEP level associated with an RRI ≥ 0.70 was associated with subsequent development of AKI (OR = 1.567 [95% CI 1.173–2.095; *p* < 0.001). The relationship between AKI and the difference between the clinical PEEP and the PEEP level associated with RRI ≥ 0.70 during the PEEP trial is summarized in Fig. 3. This aspect should be considered carefully from the clinical point of view. Indeed, our study suggests that the PEEP level may have a detrimental effect on kidney function. This suggests that, among the numerous physiological effects of PEEP, a “personalized” PEEP setting approach should also consider kidney protection. This issue may gain clinical relevance when trying to combine lung protection and maintenance of kidney function. It is tempting to speculate that monitoring RRI during a PEEP trial could be a tool to identify ventilated patients at risk of AKI and the PEEP level potentially able to induce AKI, but our study design does not allow to draw any conclusion and more studies are needed.

Our study has several limitations. First, it is a single center study and thus the results may reflect local practice. Despite promising initial reports have demonstrated that RRI could be a useful tool to evaluate kidney perfusion, recent conflicting results have emerged from recent multicenter studies, prompting the need for other studies to clarify which other factors (i.e. PEEP) may influence the application of RRI in the clinical practice. Higher RRI, indeed, is not solely attributable to ventilation and the higher RRI in older patients and with hypertension may have contribute to a higher incidence of AKI.

Second, our observational design does not allow to speculate whether bedside RRI evaluation can carry any advantage on clinical outcomes. Third, we did not register intra-abdominal pressure at each level of PEEP, which may directly affect renal blood flow and thus, we cannot exclude that the PEEP impact on of RRI was mediated by a PEEP-induced increase in intra-abdominal pressure. Fourth, we only measured the RRI in one side and we did not measure the renal venous stasis index [27], renal venous flow pattern or the vena cava diameter which could add some data regarding the relationship between PEEP and renal congestion.

## Conclusions

In conclusion, the RRI seems a valuable tool to assess the risk of AKI in mechanical ventilated patients at the bedside; further, repeated RRI measurement at different levels of PEEP may help to assess the impact of PEEP on renal hemodynamics.

## Electronic supplementary material

Below is the link to the electronic supplementary material.

Supplementary Material 1

Supplementary Material 2

Supplementary Material 3

Supplementary Material 4

Supplementary Material 5

## Data Availability

Data can be shared under reasonably request.

## Abbreviations

ICU: intensive care unit
AKI: acute kidney injury
GFR: glomerular filtration rate
PEEP: positive end-expiratory pressure
RRI: renal resistive index
SAPS: Simplified Acute Physiology Score
BGA: Blood gas analysis
ΔP: driving pressure
KDIGO: Kidney Disease: Improving Global Outcomes
CO: cardiac output
LVOT: left ventricular outflow tract
VTI: velocity time integral
CVP: central venous pressure
