# Supplementary figures and images for: Impact of positive end-expiratory pressure on renal resistive index in mechanical ventilated patients
Source: J Clin Monit Comput. 2024 May 21;38(5):1145–53. doi: 10.1007/s10877-024-01172-z (PMC11427533; doi:10.1007/s10877-024-01172-z)

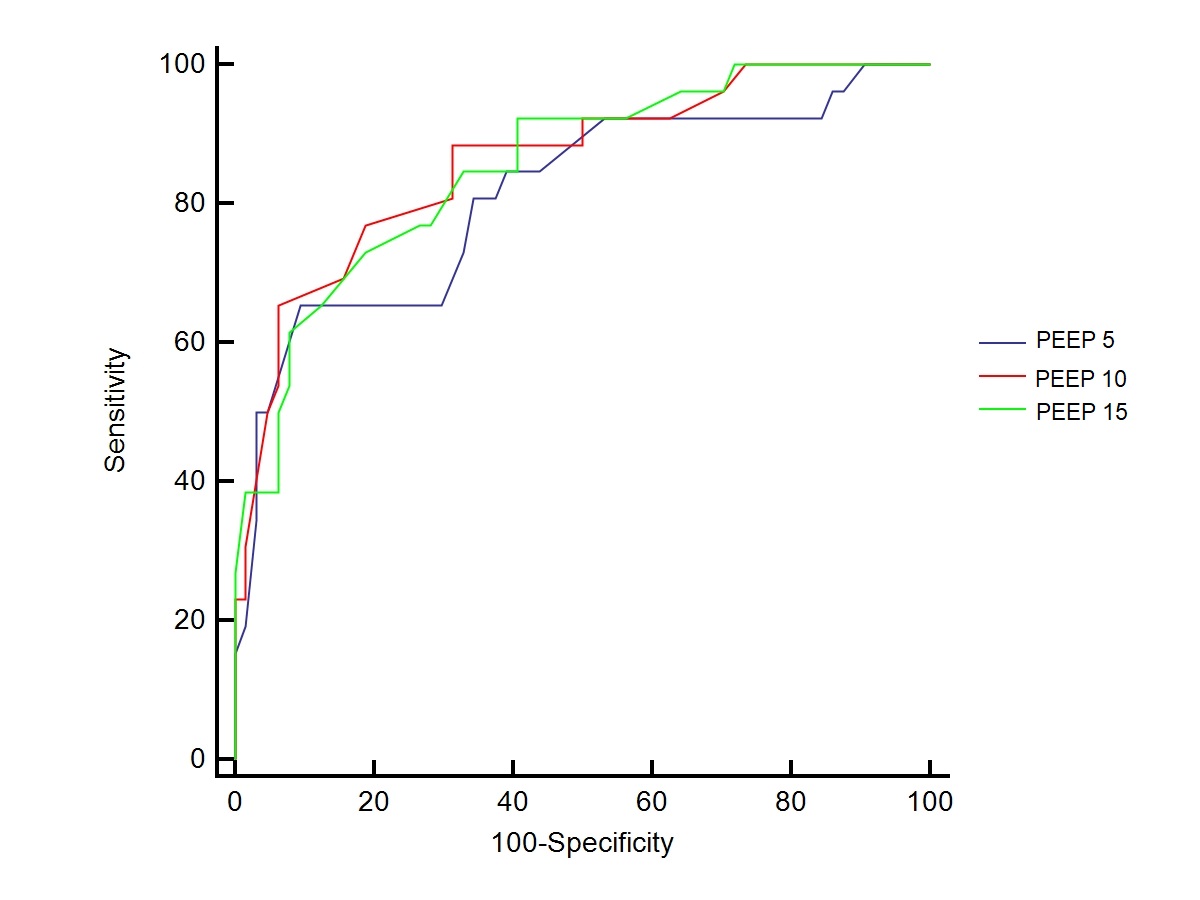

Supplement: Supplementary file 2 — Supplementary Material 2 [file 10877_2024_1172_MOESM2_ESM.jpg]

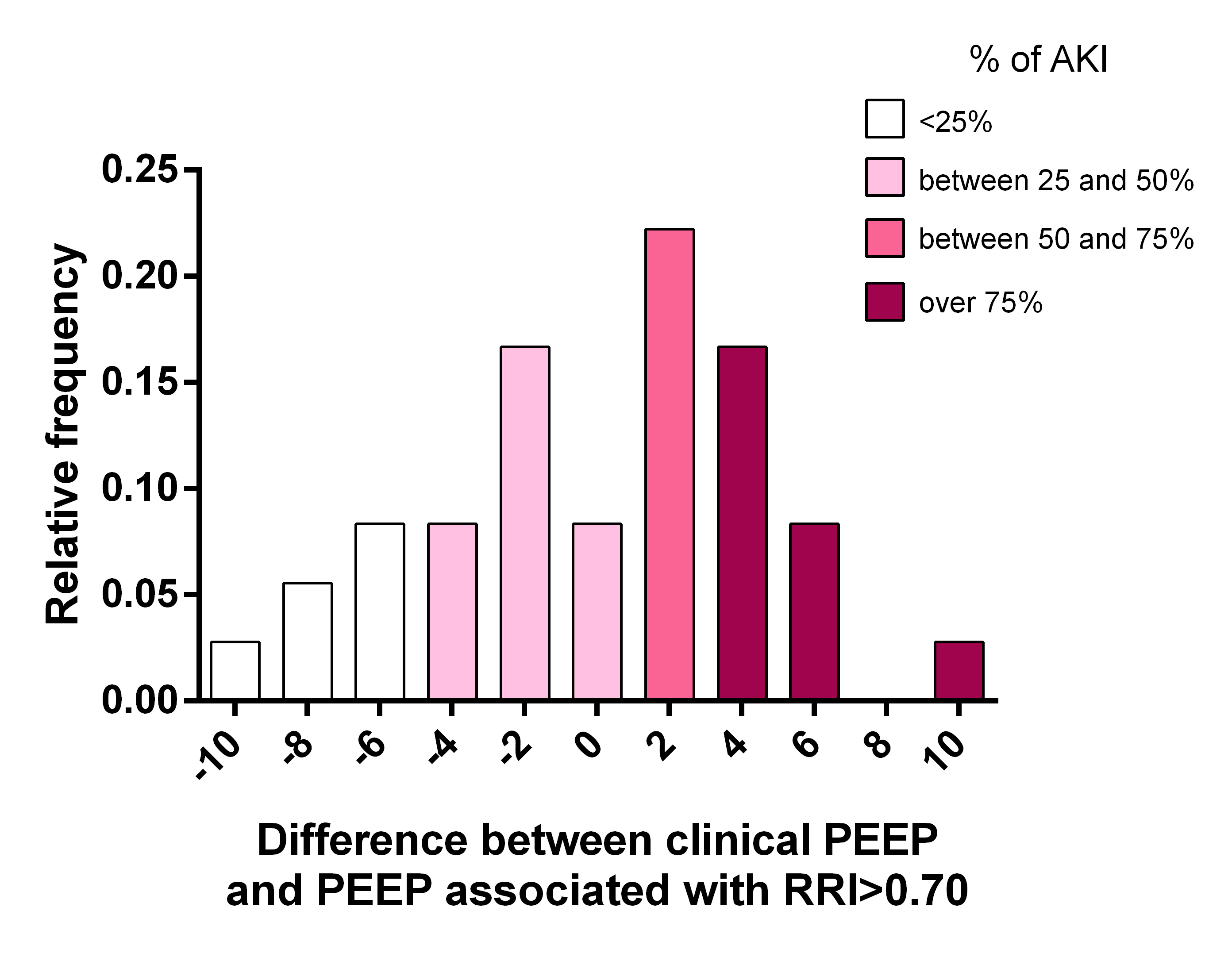

Supplement: Supplementary file 3 — Supplementary Material 3 [file 10877_2024_1172_MOESM3_ESM.jpg]

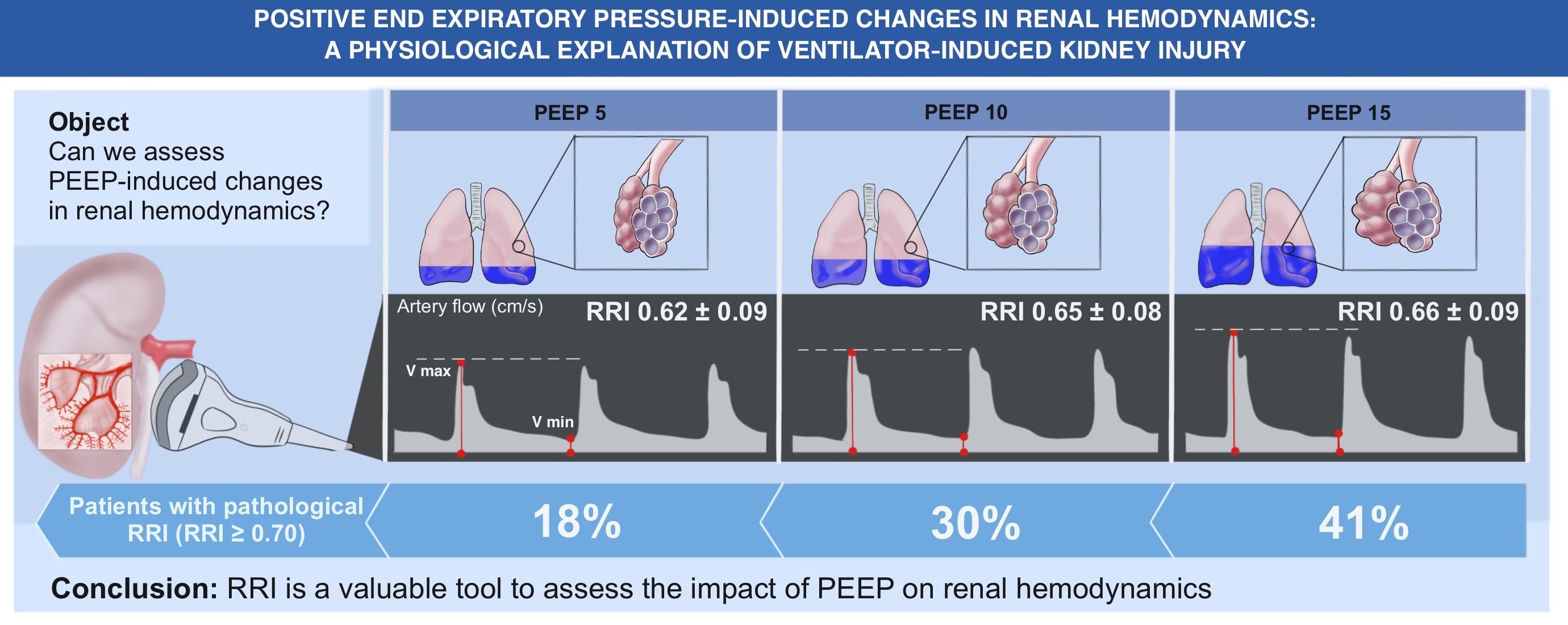

Supplement: Supplementary file 5 — Supplementary Material 5 [file 10877_2024_1172_MOESM5_ESM.jpg]
