# Supplementary material for: Impact of positive end-expiratory pressure on renal resistive index in mechanical ventilated patients
Source: J Clin Monit Comput. 2024 May 21;38(5):1145–53. doi: 10.1007/s10877-024-01172-z (PMC11427533; doi:10.1007/s10877-024-01172-z)
Supplement: Supplementary file 4 — Supplementary Material 4 [file 10877_2024_1172_MOESM4_ESM.doc]

Patients expected to be mechanically ventilated for at least 48 hours (n=185)

(

Excluded (n=80)

- Arrhythmia (n=45)

- Presence of an aortic prothesis (n=15)

- Renal replacement therapy before study inclusion (n=19)

- Pregnancy (n=1)

105 patients included

Lost to follow up (n=13)

- Unsatisfying ultrasound imaging (n=10)

- Hemodynamic instability (n=3)

92 patients analysed

**RRI ability to predict AKI at each level of PEEP**

Patients with MMSE < 26 (n 5)

PEEP 5: AUROC 0.812 [95%CI 0.705 – 0.918]

PEEP 10: AUROC 0.860 [95%CI 0.773 – 0.947]

PEEP 15: AUROC 0.853 [95% CI 0.767 – 0.939]
